# Local connections among excitatory neurons underlie characteristics of enriched environment exposure-induced neuronal response modulation in layers 2/3 of the mouse V1

**DOI:** 10.3389/fnsys.2025.1525717

**Published:** 2025-02-19

**Authors:** Nobuhiko Wagatsuma, Yuka Terada, Hiroyuki Okuno, Natsumi Ageta-Ishihara

**Affiliations:** ^1^Department of Information Science, Faculty of Science, Toho University, Chiba, Japan; ^2^Department of Biomolecular Science, Faculty of Science, Toho University, Chiba, Japan; ^3^Laboratory of Biochemistry and Molecular Biology, Graduate School of Medical and Dental Sciences, Kagoshima University, Kagoshima, Japan

**Keywords:** enriched environmental exposure, cortical microcircuit model, layers 2/3 of V1, mouse, c-Fos, log-normally distributed synaptic weight

## Abstract

Environmental enrichment, an enhancement in the breeding environment of laboratory animals, enhance development of the cortical circuit and suppresses brain dysfunction. We quantitatively investigated the influences of enriched environment (EE) exposure, on responses in layers 2/3 (L2/3) of the primary visual area (V1) of mice. EE modifies visual cortex plasticity by inducing immediate early genes. To detect this, we performed immunostaining for the immediate early gene product c-Fos. EE exposure significantly increased the number of neurons with high c-Fos fluorescence intensity compared with those of mice under standard housing (SH). In contrast, there was no significant difference in the number of neurons exhibiting low c-Fos intensity between the SH and EE exposure groups. To further investigate the mechanism of modulation by EE exposure, we developed a microcircuit model with a biologically plausible L2/3 of V1 that combined excitatory pyramidal (Pyr) neurons and three inhibitory interneuron subclasses. In the model, synaptic strengths between Pyr neurons were determined according to a log-normal distribution. Model simulations with various inputs mimicking physiological conditions for SH and EE exposure quantitatively reproduced the experimentally observed activity modulation induced by EE exposure. These results suggested that synaptic connections among Pyr neurons obeying a log-normal distribution underlie the characteristic EE-exposure-induced modulation of L2/3 in V1.

## 1 Introduction

A diverse range of stimuli from the external world is crucial for animal brains, not only to establish various perceptions and understand the real world (Hubel and Wiesel, 1968; Zhou et al., 2000; Pasupathy and Connor, 2002; Schmehl et al., 2024) but also to develop the neuronal circuits necessary to process this information (Ishikawa et al., 2014; Jenks and Shepherd, 2020). Environmental enrichment, which enhance the breeding environment for laboratory animals, improves the development of the cortical circuit for information processing while also suppressing brain dysfunction and anxiety disorders (Cancedda et al., 2004; Kempermann, 2019; Bibollet-Bahena et al., 2023). Previous studies have reported that enriched environment (EE) exposure exerts a broad range of effects on the brain, including enhanced activation (Sampedro-Piquero et al., 2015), modulation of interactions between cortical areas (Garbo et al., 2011), and improved cognitive capabilities (van Praag et al., 2020; Sale et al., 2009; Mainardi et al., 2010; Miranda et al., 2024). To understand the detailed neuronal mechanisms of information processing and devise methods to improve neuronal system dysfunction, it is crucial to comprehend how EE exposure, by increasing the variety of stimuli from the external world, modulates brain activities.

The primary visual cortex (V1) is one of the most well-studied targets of EE exposure (Bibollet-Bahena et al., 2023; Garbo et al., 2011; Kalogeraki et al., 2017). V1 microcircuits consist of a six-layered network structure, based on excitatory pyramidal (Pyr) neurons and inhibitory interneurons (Binzegger et al., 2004; Thomson and Morris, 2002; Thomson et al., 2002; Potjans and Diesmann, 2014). The superficial layers 2/3 (L2/3) in the layered microcircuit play a critical role in organizing visual perception and understanding the external world by integrating various neuronal signals, including feedforward inputs representing visual stimuli, feedback signals from higher visual areas, and horizontal projections from surrounding microcircuits (Gilbert and Wiesel, 1983; Self et al., 2013; van Kerkoerle et al., 2014). Prior studies have reported that the neural circuit in L2/3 of V1 is mainly constructed based on Pyr neurons and three subclasses of inhibitory interneurons: parvalbumin (PV), somatostatin (SOM), and vasoactive intestinal polypeptide (VIP). Recently, physiological and computational studies have begun to elucidate the details of the neural circuit structure and the interactions among these neuron classes and subclasses (Zhang et al., 2014; Neske et al., 2015; Mardinly et al., 2016; Cardin, 2018; Lee et al., 2017; Lee et al., 2018; Ito et al., 2024; Jiang et al., 2024). However, the characteristics and detailed mechanisms of modulation of neuronal activities in L2/3 of V1 through EE remain unclear.

In this study, we conducted a quantitative analysis to investigate the impact of the first exposure to EE, on neuronal responses in L2/3 of the mouse V1. EE alters the plasticity of the visual cortex through induction of immediate early genes (Montero, 1997; Cancedda et al., 2004; Piché et al., 2004). To identify the expression of immediate early genes following EE exposure, we conducted immunostaining for the immediate early gene product c-Fos (Sheng and Greenberg, 1990). We observed a significant increase in the number of neurons in L2/3 exhibiting high c-Fos fluorescence intensity under the EE exposure condition compared with those from mice under the standard housing (SH) condition. However, the number of neurons representing low c-Fos intensity did not significantly differ between the SH and EE exposure conditions. The characteristic modulation patterns observed in L2/3 of the mouse V1 through physiological experiments were reproduced by a cortical microcircuit model based on the biological architecture of L2/3 of the V1. These physiological and computational results indicated the modulatory effects of the switch from SH to EE on neuronal activities in L2/3 of the V1 and suggested the neural circuit structures responsible for inducing such modulation.

## 2 Materials and methods

### 2.1 Physiological experiments

#### 2.1.1 Animal experiments and the switch from SH to EE

**Figure 1:**
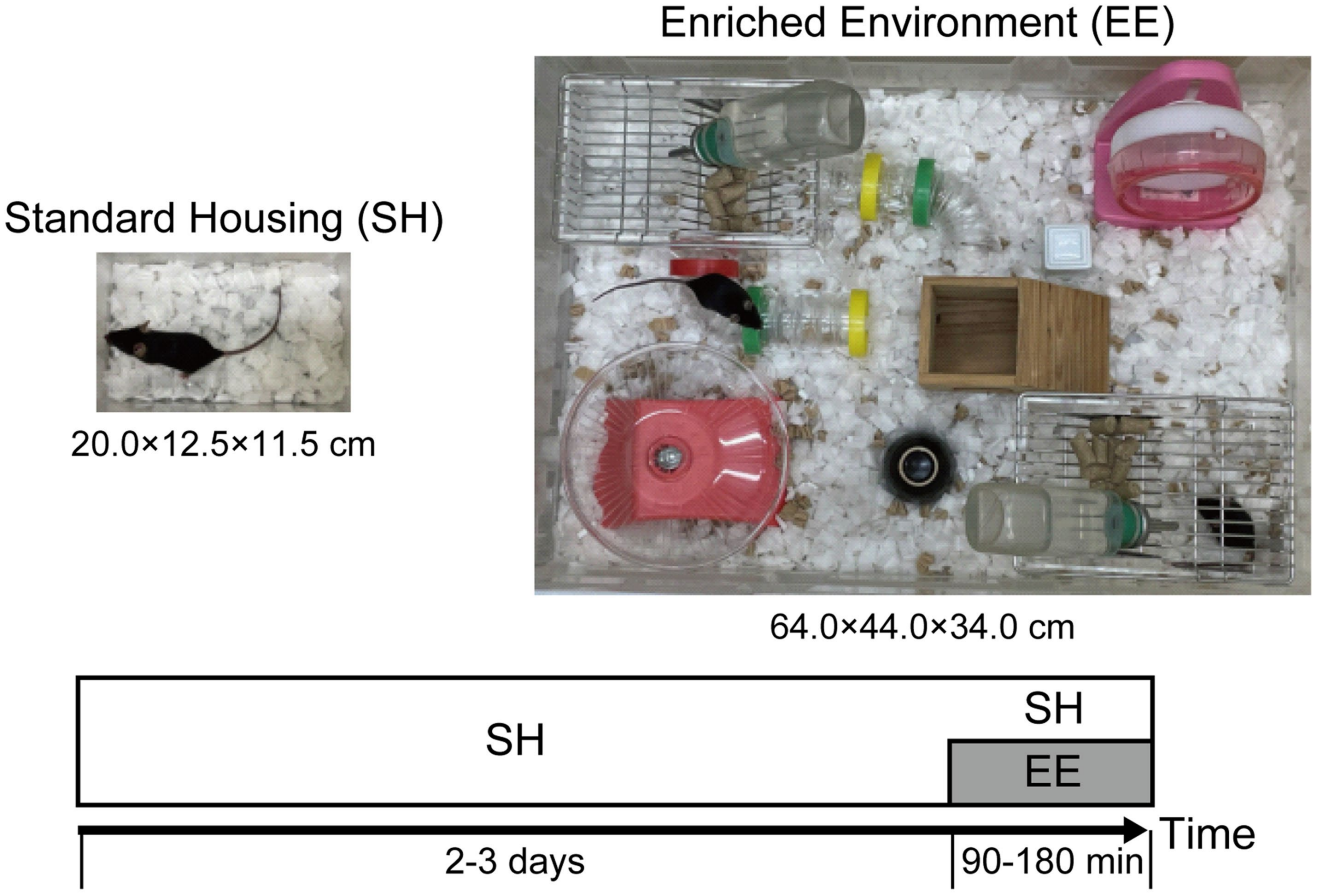
Experimental design to investigate the modulation of neuronal response in layers 2/3 (L2/3) of the primary visual cortex (V1) induced by the switch from standard housing (SH) to enriched environment (EE) exposure. Experimental procedures and design to measure neuronal activity in mouse L2/3 of V1. Mice housed in individual cages (20.0 × 12.5 × 11.5 cm) for 2–3 days were divided into the SH and EE exposure conditions. Under the EE exposure condition, in larger cages (64.0 × 44.0 × 34.0 cm), the mice were allowed to be active in an environment that enhances cognitive, physical, and social stimulation for 90–180 min.

All experiments were performed in accordance with the guidelines of the National Institutes of Health (1996) and Japan Neuroscience Society and approved by Toho University Animal Care and Use Committee (Approval No. 22-519). C57BL/6N strain wild-type male mice (7 weeks old) were purchased from Japan SLC, Inc. Mice were housed in a conventional facility with a 12-h light/dark cycle (light period from 6:00 to 18:00), temperature of 22 ± 2°C, and free access to food and water in individual cages (*W* × *D* × *H* = 20 × 12.5 × 11.5 cm, Figure 1). Based on the protocol described in the previous study (Kawashima et al., 2009), the animals were kept for 2–3 days at a light intensity of 300 lux in the cage during the light period. The switch from SH to EE, EE exposure, was performed in different large cages (*W* × *D* × *H* = 64 × 44 × 34 cm, Figure 1) including a wider variety of objects between 9:00 and 15:00 for 90–180 min, with food and water available ad libitum, and the light inside the apparatus was adjusted to 300 lux. In contrast, mice that were individually housed in their home cages were designated as the SH group.

#### 2.1.2 Immunohistochemical staining

Mice were anesthetized and transcardially perfused with 4 mL of 25 mM phosphate buffered saline (PBS) chilled to 4°C and then with 50 mL of 2% paraformaldehyde (PFA) in 0.1 M phosphate buffer (PB) at a flow rate of 4.2 mL/min. After perfusion, brains were extracted and post-fixed in 2% PFA/PB at 4°C overnight. The brains were cryoprotected with 15% sucrose/PBS for 12 h, followed by 30% sucrose/PBS for 12 h. The brains were then embedded in OCT compound (Tissue-Tek) and coronal brain sections of 30 μm thickness were prepared using a microtome (REM-700, Yamato Kohki Industrial). Sections were collected at −3.16 mm from Bregma, referencing a brain atlas. The sections were washed three times in 25 mM PBS for 5 min each. After washing with PBS-T (PBS containing 0.3% Triton X-100), the sections were blocked in blocking buffer (5% normal goat serum/1% bovine serum albumin/0.3% Triton X-100 in PBS) at room temperature for 60 min. The sections were then incubated with rabbit anti-c-Fos antibody (1:1,000, 2,250, CellSignaling) in blocking buffer for 2 days at 4°C. Next, the sections were washed three times with PBS-T and then incubated with the secondary antibody (anti-rabbit Alexa-555 antibody (1:1,000, A-21429, Invitrogen) in 5% normal goat serum/PBS) for 2 h at room temperature. After three washes in PBS-T, the sections were stained with DAPI (1:1,500, D1306, Molecular Probes), washed twice more in PBS-T, fixed for 5 min in 4% PFA/PBS, followed by two final washes in PBS-T, and mounted using anti-fade mounting medium (mounting medium, vector). As described in a previous paper, among methods to detect immediate early genes, c-Fos antibodies are most widely used for immunohistochemical staining of mouse brain sections (Pompeiano and Colonnese, 2023).

#### 2.1.3 Image acquisition and data analysis of neuronal responses in mouse V1

Confocal images of the brain tissue were obtained using a confocal laser microscope (LSM900 with Airyscan 2, Carl Zeiss) with a 20× objective lens (Plan-Apochromat NA 0.8). L2/3 of the V1 was defined as a region of interest (ROI) using DAPI fluorescence as an indicator, from which the number of DAPI-positive cells and the area of the analysis region were determined. Subsequently, we counted the number of c-Fos-positive cells within the ROI. In addition, we obtained the number of cells with high c-Fos fluorescence intensity (c-Fos-strong positive cells) from these images by subtracting the 2-fold background intensity from these images. To determine the number of cells with low c-Fos fluorescence intensity (c-Fos-weak positive cells), the number of c-Fos-strong positive cells was subtracted from the total number of c-Fos positive cells with c-Fos fluorescence intensity from these images by subtracting the background intensity from these images.

#### 2.1.4 Statistical analyses of animal experiments

Quantitative data are represented as the median or mean ± standard error of the mean. Statistical analyses were conducted using Prism (GraphPad Software). A *t*-test was applied for comparisons between two groups.

### 2.2 Computational model and simulations

#### 2.2.1 Cortical microcircuit model of L2/3 of V1

**Figure 2:**
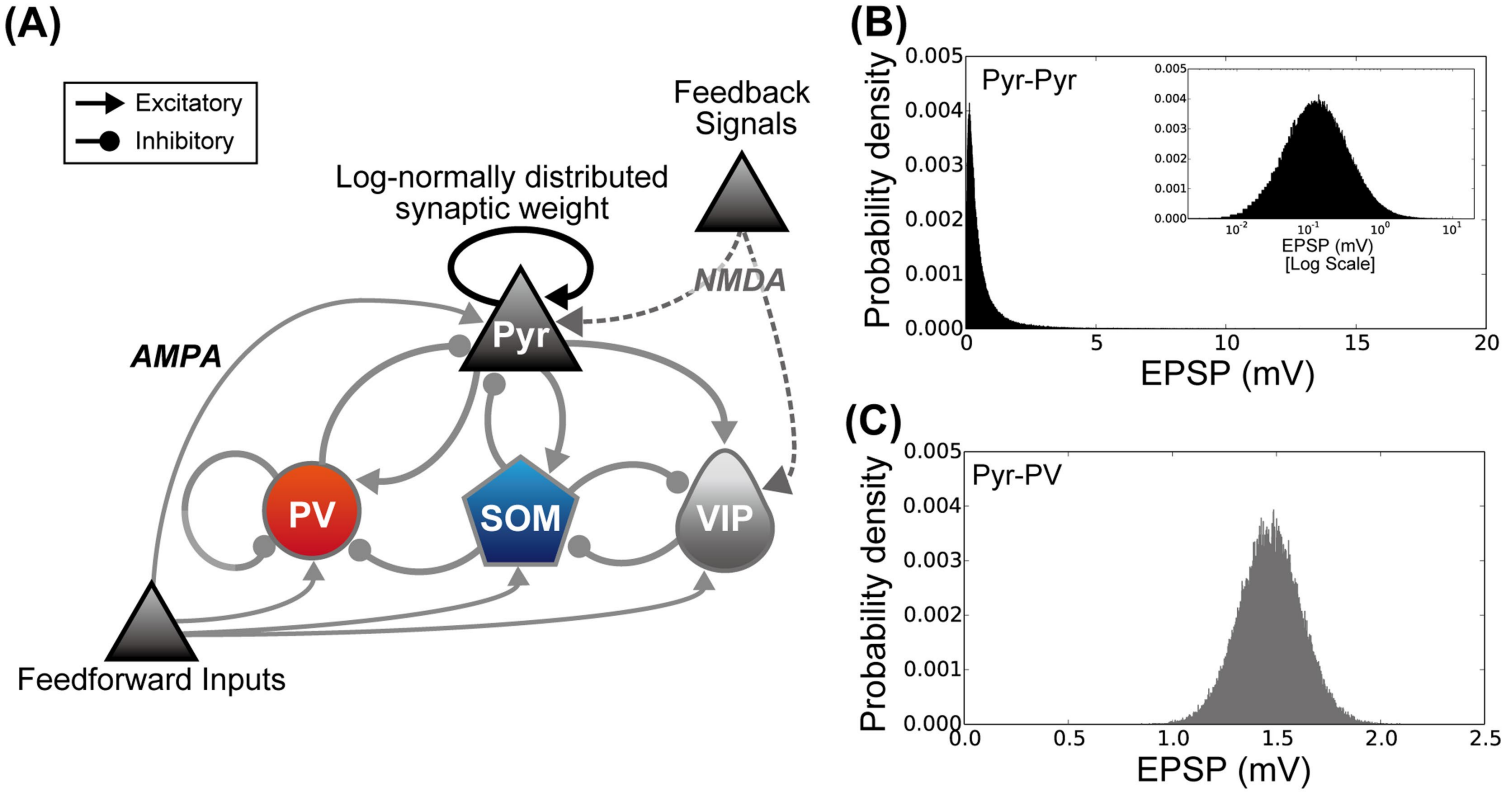
The microcircuit used to simulate the activity of layers 2/3 of the primary visual cortex under the standard housing and enriched environment exposure conditions. **(A)** Architecture of the proposed cortical microcircuit model. The model network was designed according to our prior computational model (Wagatsuma et al., 2023). Triangular and circular arrowheads represent excitatory and inhibitory connections, respectively. Connections between pyramidal (Pyr) model neurons (black triangular arrowheads) indicate that synaptic connections are distributed according to a log-normal distribution. The synaptic weights of other connections obeyed a Gaussian distribution (gray triangular and circular arrowheads) (Song et al., 2005; Lefort et al., 2009; Teramae et al., 2012). **(B)** The distribution of synaptic weights among Pyr model neurons. Excitatory postsynaptic potentials from Pyr to Pyr model neurons follow a log-normal distribution [black triangular arrowheads in the panel **(A)**]. The inset graph is a plot of the same distribution using a log scale for the *x*-axis. **(C)** Example distribution of synaptic weights from Pyr model neurons to model inhibitory interneurons (from Pyr to parvalbumin (PV) populations). In contrast to connections between Pyr model neurons, the synaptic weights from excitatory to inhibitory, inhibitory to inhibitory, and inhibitory to excitatory neurons are distributed according to a Gaussian distribution [gray triangular and circular arrowheads in the panel **(A)**].

To investigate the neural mechanism of modulation by switching from SH to EE, we performed simulations of the cortical microcircuit model of L2/3 of V1, which was based on excitatory Pyr neurons and three subclasses of inhibitory interneurons (PV, SOM, and VIP). The network structure is illustrated in Figure 2A. The scale and structure of the model network were the same as those used in our previous computational study (Wagatsuma et al., 2023). This network structure was derived from prior circuit models reported by physiological and computational studies (Lee et al., 2017; Lee et al., 2018; Teramae et al., 2012; Pfeffer et al., 2013; Nobukawa et al., 2022). In the following section, we describe the essential elements of our microcircuit model. For a full description of our model, we refer the reader to our previous study (Wagatsuma et al., 2023).

The full network of our model consisted of approximately 2,650 integrate-and-fire model neurons (2,068 Pyr, 268 PV, 175 SOM, and 140 VIP neurons) and approximately 900,000 model synapses. These were determined according to previous computational studies (Potjans and Diesmann, 2014; Lee et al., 2017). The number of synapses was determined according to the connection probability (see Wagatsuma et al., 2023 for details). The dynamics of the membrane potential (*V*) of integrate-and-fire model neurons were determined by the following equation:


(1) $$\begin{array}{l} \frac{dV\left(t\right)}{dt}=-\frac{V\left(t\right)-E_{l}}{\tau_{m}}\\ \;+\;\frac{I_{Pyr}\left(t\right)+I_{PV}\left(t\right)+I_{SOM}\left(t\right)+I_{VIP}\left(t\right)+I_{ext}\left(t\right)}{C_{m}}, \end{array}$$

where the membrane time constant, $\tau_{m}$, was chosen depending on the neuron class and inhibitory interneuron subclass. In this study, we used 10.5, 3.1, 11.8, and 10.9 ms for Pyr neurons and PV, SOM, and VIP interneurons, respectively. In contrast, the membrane capacitance, $C_{m}$, was set to 200 pF irrespective of the neuron class or subclass. The leak reversal potential was $E_{l}=-70\;\text{mV}$.

The synaptic currents, *I*_Pyr_(*t*), *I*_PV_(*t*), *I*_SOM_(*t*), and *I*_VIP_(*t*), flowing into the model neuron from Pyr neurons and the PV, SOM, and VIP interneuron subclasses were determined by the following equation (Buehlmann and Deco, 2008; Deco and Thiele, 2011; Wagatsuma et al., 2022; Wagatsuma et al., 2023):


(2) $$I_{syn}\left(t\right)=g_{j}^{syn}\left(V\left(t\right)-V_{NC}\right)\sum_{j}s_{j}^{syn}\left(t\right),$$

where *V*_NC_ indicates the reversal potential depending on the model neuron class. We used $V_{NC}=0\;\text{mV}$ for *I*_Pyr_(*t*) for the excitatory synaptic current. To implement inhibitory synaptic currents for *I*_PV_(*t*), *I*_SOM_(*t*), and *I*_VIP_(*t*), $V_{NC}$ was set to −70 mV. The conductance strength of the fully activated synapse, *g*^syn^, was determined by classes and subclasses of pre- and postsynaptic neurons. In this study, the strength and the distribution of *g*^syn^ were chosen according to our prior computational model (Wagatsuma et al., 2023). The *g*^syn^ strengths among Pyr neurons were determined using a log-normal distribution (Figure 2B) (Song et al., 2005; Lefort et al., 2009; Teramae et al., 2012; Nobukawa et al., 2022), whereas other synaptic connections obeyed a Gaussian distribution (Figure 2C). The mean and variance for the *g*^syn^ strengths were the same as those used in our prior computational study (Wagatsuma et al., 2023). $s_{j}^{syn}$ represented the fraction of open channels from the *j*-th model neuron, which was computed as:


(3) $$\frac{ds_{j}^{syn}}{dt}=-\frac{s_{j}^{syn}\left(t\right)}{\tau_{syn}}+\sum_{k}\delta \left(t-t_{j}^{k}-d_{j}\right),$$

where $\tau_{syn}$ represents the time constant for the post-synaptic decay, which was determined by the connections from pre- and post-synaptic neuron classes (Pfeffer et al., 2013; Lee et al., 2018; Wagatsuma et al., 2023). The sum over *k* ran over all spikes from connecting presynaptic model neurons. Each spike was represented by a Dirac delta function, $\delta \left(t\right)$, which assumes a nonzero value at the spike times ($t_{j}^{k}$) of the presynaptic neurons (and zero at all other times) and integrates to one over any interval encompassing $t_{j}^{k}$. *d_j_* is the delay from the *j*-th presynaptic neuron. The synaptic delay from the *j*-th presynaptic neuron, *d_j_*, was determined by a Gaussian distribution with a mean of 2.0 ms and a variance of 0.2 ms for excitatory connections and a mean of 1.0 ms and a variance of 0.1 ms for inhibitory connections. These means and variances for the inhibitory synaptic delay were applied irrespective of the interneuron subclass.

*I*_ext_(*t*) in Equation 1 represents the synaptic currents of external inputs to model neurons. Our model received three types of external inputs: background inputs to induce spontaneous activity, feedforward inputs from the retina, and feedback signals from higher visual cortices such as V2 and V4. These external inputs were given by independent Poisson spike trains. All model neurons received the background inputs. Feedforward inputs were directed to their target model neuron populations, selected randomly based on the connection probabilities. The background and feedforward inputs were mediated by AMPA type glutamatergic receptors. In contrast, feedback signals were projected to Pyr neuron and VIP interneuron populations, which were given by NMDA receptor-mediated synaptic currents. These feedforward inputs driven by the AMPA and feedback signals given by NMDA receptors were consistent with previous physiological studies of V1 (Self et al., 2012; Herrero et al., 2013). Additionally, previous computational studies have suggested that feedback signals mediated by NMDA synapses contribute to the modulation of both neuronal activity and the synchronization of responses in the visual cortex (Wagatsuma et al., 2016; Wagatsuma et al., 2021). The populations used to represent these feedforward inputs and feedback signals both consisted of 100 Poisson spike neurons. The connection probability for feedforward inputs was determined according to our prior computational model (Wagatsuma et al., 2023). The connection probability from feedback signals to the populations of Pyr neurons and VIP interneurons were 0.075 and 0.05, respectively.

The synapses used to model the external inputs in this study were the same as those used by our prior computational model (Wagatsuma et al., 2023). The AMPA type glutamatergic receptors mediating background and feedforward inputs were defined as:


(4) $$I_{\text{AMPA}}\left(t\right)=g_{j}^{\text{AMPA}}\left(V\left(t\right)-V_{E}\right)\sum_{j}s_{j}^{\text{AMPA}}\left(t\right),$$

where $g^{\text{AMPA}}$ is the conductance of the fully activated synapse for background or feedforward inputs. *V*_E_ = 0 mV represents the reversal potential of the excitatory synapse. The fraction of open channels of model neurons from the *j*-th Poisson spike train ($s_{j}^{\text{AMPA}}$) was determined as:


(5) $$\frac{ds_{j}^{\text{AMPA}}}{dt}=-\frac{s_{j}^{\text{AMPA}}\left(t\right)}{\tau_{\text{AMPA}}}+\sum_{k}\delta \left(t-t_{j}^{k}-d_{j}\right),$$

where the postsynaptic-decay time constant for background and feedforward inputs is $\tau_{\text{AMPA}}$ = 2.0 ms, irrespective of the class of target neuron.

In this study, we used a standard computational model for generic NMDA receptors (Wang, 1999), where the NMDA-receptor mediating synaptic current, *I*_NMDA_, was defined as follows:


(6) $$I_{\text{NMDA}}\left(t\right)=\frac{g_{j}^{\text{NMDA}}\left(V\left(t\right)-V_{E}\right)}{1+\left[{\text{Mg}}^{2+}\right]\exp\left(-0.062V\left(t\right)\right)/3.57}\sum_{j}s_{j}^{\text{NMDA}}\left(t\right),$$

where $g^{\text{NMDA}}$ is the synaptic conductance of a fully open NMDA synapse. [Mg^2+^] = 1 mM represents a voltage-dependent block of NMDA channels (Jahr and Stevens, 1990). The fraction of open NMDA channels in a synapse is $s_{j}^{\text{NMDA}}$, which was calculated as:


(7) $$\frac{ds_{j}^{\text{NMDA}}\left(t\right)}{dt}=-\frac{s_{j}^{\text{NMDA}}\left(t\right)}{\tau_{\text{NMDA},\text{decay}}}+\alpha x\left(t\right)\left(1-s_{j}^{\text{NMDA}}\left(t\right)\right),$$

(8) $$\frac{dx\left(t\right)}{dt}=-\frac{x\left(t\right)}{\tau_{\text{NMDA},\text{rise}}}+\sum_{k}\delta \left(t-t_{\text{NMDA}}^{k}\right),$$

where *α* = 1/ms. The rise time for an NMDA synapse is $\tau_{\text{NMDA},\text{rise}}$ = 2 ms, and their decay time constant is $\tau_{\text{NMDA},\text{decay}}$ = 100 ms (Buehlmann and Deco, 2008; Wagatsuma et al., 2023). The sum over *k* is over spike time ($t_{\text{NMDA}}^{k}$), which, here, is the times at which spikes occur in the Poisson spike train representing the feedback signals.

The experimental conditions for SH and EE exposure were determined by the strengths of feedforward inputs and feedback signals. Under the EE exposure condition, a wider variety of visual objects were projected onto the animals’ visual field to activate neurons across different levels of the visual cortex compared with that in the SH condition. Furthermore, EE exposure may enhance interactions between animals and objects within their home cage, as well as increase locomotion in animals. We speculated that under the EE exposure condition, both feedforward inputs and feedback signals are more actively projected onto the cortical microcircuit in V1 than those under the SH condition. In this study, to simulate the SH condition, we employed Poisson spike trains of 10 Hz for the feedforward inputs and 5 Hz for the feedback signals. Conversely, in the EE exposure condition, the firing rate of the feedforward inputs was increased to 20 Hz. Similarly, the neurons representing the feedback signals in the EE exposure condition exhibited a firing frequency of 10 Hz.

#### 2.2.2 Computational simulations and numerical experiments of our proposed model

The differential equations presented in Equations 1–8 were integrated by employing a fourth-order Runge–Kutta algorithm with a time step of 0.1 ms. The simulation code was written in the C programing language.

To investigate the mechanism of modulation by the switch from SH to EE, we performed simulations of the proposed microcircuit model using external inputs mimicking the SH and EE exposure conditions. As shown previously, these conditions were determined by the strengths of the feedforward inputs and the feedback signals. We conducted 10 trials of the model simulations, each lasting 60 biological seconds, under these conditions. The simulation length and the number of trials were determined based on the scale of the model network and computational cost constraints.

#### 2.2.3 Data analysis of microcircuit model responses

To relate the activities of our cortical microcircuit model to the neuronal responses experimentally observed in L2/3 of the mouse V1, we computed the firing rates of individual Pyr model neurons under both the SH and EE exposure conditions. In this study, we assumed that Pyr model neurons with a firing rate ranging from 1.5 to 3.0 Hz correspond to c-Fos-weak positive cells (weak positive Pyr). In contrast, Pyr model neurons exhibiting a firing rate exceeding 3.0 Hz were classified as c-Fos-strong positive cells (strong positive Pyr). In addition, we computed the ratios of weak and strong positive Pyr neurons within the population of Pyr model neurons to relate to the neuronal responses in L2/3 of the mouse V1 to the activities of our model. For the statistical analysis, we applied a *t*-test to the responses of Pyr model neurons, following to the animal experiments.

## 3 Results

### 3.1 Modulation of neurons in L2/3 of the mouse V1 induced by the switch from SH to EE

**Figure 3:**
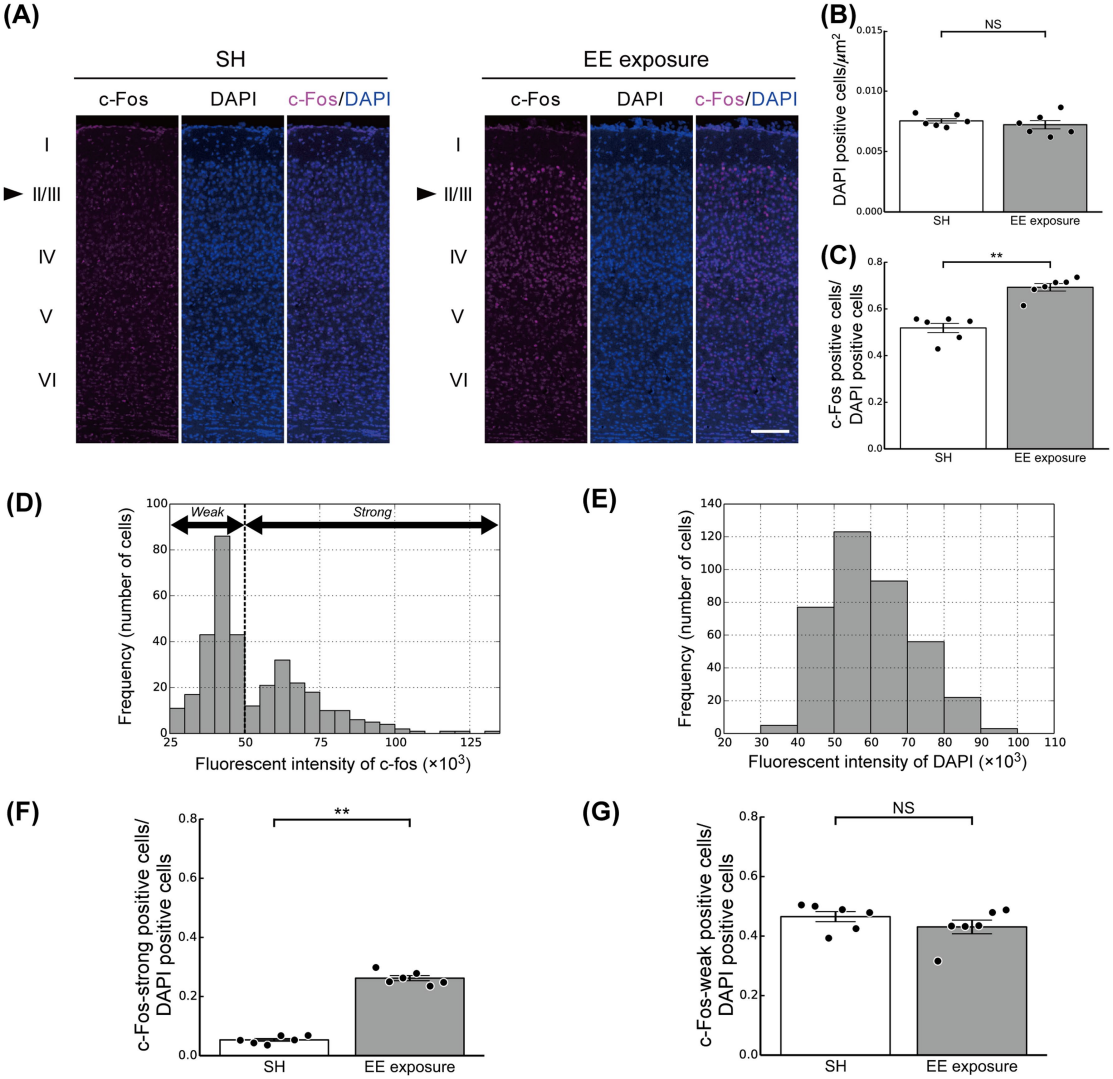
Enriched environment (EE) exposure-induced modulation of the ratios of c-Fos-positive cells in layers 2/3 (L2/3) of the mouse primary visual cortex (V1). **(A)** Representative images of coronal sections of V1 under the standard housing (SH) (left panel) and EE exposure (right panel) conditions. The sections were stained with a c-Fos antibody (left) and DAPI (center) in each panel. Neuronal response was determined by the fluorescence intensity. Arrowheads indicate L2/3. Scale bars, 100 μm. **(B)** The ratio of DAPI-positive cells in L2/3 of the mouse V1 under the SH (white bar) and EE exposure (gray bar) conditions. **(C)** Ratios of c-Fos-positive cells in L2/3 of the mouse V1 under the SH and EE exposure conditions. **(D)** The distribution of c-Fos fluorescence intensity. **(E)** The distribution of DAPI fluorescence intensity. **(F)** Ratio of c-Fos-strong positive cells. **(G)** Ratio of c-Fos-weak positive cells. Black dots represent the responses of individual animals. Error bars represent the standard error of the mean. Asterisks indicate significant differences between the two conditions (*t*-test, ^**^p < 0.01, NS, not significant).

We physiologically and computationally investigated and analyzed the impacts of the switch from SH to EE on modulation of neuronal responses in L2/3 of the mouse V1 (Figure 1). c-Fos is the protein product of the immediate early gene c-Fos and a marker of neuronal activation in layers 2/3 neurons of the mouse V1 (Nguyen et al., 2015; Pizzo et al., 2016). Its expression reaches its peak approximately 90 min after neuronal activity (Morgan and Curran, 1991; Sheng and Greenberg, 1990) and remains elevated above baseline levels until 180 min after neuronal activity (Zangenehpour and Chaudhuri, 2002). Mice were exposed to EE, and immunohistochemical staining for c-Fos, was performed 90 to 180 min (Figure 3A) after the switch from SH to EE.

Quantification of cells in L2/3 of V1 using DAPI staining (nuclear staining; the center panel in Figure 3A) revealed no difference in the number of cells per unit area between the SH and EE exposure groups (*t*-test, *p* = 0.47; Figure 3B). To further investigate the impact of the switch from SH to EE on modulation of neuronal responses, we analyzed the number and distribution of c-Fos-positive neurons in L2/3 of V1 under both the SH and EE exposure conditions (Figures 3C,D). In this study, c-Fos positive cells were identified based on c-Fos fluorescence intensity that was higher than the background intensity (see also the Materials and Methods section for details). We used the fluorescence intensity as the metric for the neuronal responses. The distributions of c-Fos and DAPI fluorescence intensities were shown in Figures 3D,E. Calculation of the number of c-Fos-positive neurons in the L2/3 area of V1 showed an increased number in the EE exposure condition compared with that of the SH condition (*t*-test, *p* < 0.01; Figure 3C).

We further found that c-Fos-positive neurons could be divided into groups with high and low c-Fos fluorescence intensity (Figure 3D). For a more detailed analysis of the neuronal modulation induced by EE exposure, neurons with c-Fos fluorescence intensity more than twice that of the background of tissue sections were classified as neurons with high c-Fos fluorescence intensity (c-Fos-strong positive cells). In contrast, to determine the number of neurons with low c-Fos fluorescence intensity (c-Fos-weak positive cells), we subtracted the number of c-Fos-strong positive cells from the total number of c-Fos-positive cells (see also the materials and methods section for details). This threshold, defined as a c-Fos fluorescence intensity exceeding twice the background level, effectively distinguished c-Fos-strong positive cells from c-Fos-weak positive cells (Figure 3D). In contrast, no such characteristic was observed in the fluorescence intensity distribution of DAPI (Figure 3E). Then, we calculated the ratios of the c-Fos-strong and c-Fos-weak positive cells under both the SH and EE exposure conditions relative to the total number of DAPI positive cells, and the results are summarized in Figures 3F,G, respectively.

The number of c-Fos-strong positive cells under the EE exposure condition was significantly greater than that under the SH condition (*t*-test, *p* < 0.01; Figure 3F), implying that L2/3 neurons in V1 were activated by the EE exposure. However, in contrast, there was no significant difference in the ratio of c-Fos-weak positive cells between the EE exposure and SH conditions (*t*-test, *p* = 0.30; Figure 3G). These physiological findings imply a characteristic modulation pattern in L2/3 of V1 induced by the switch from SH to EE: a significant increase in the number of highly activated neurons occurs from the SH to EE exposure conditions, while the proportion of weakly activated neurons remains constant regardless of the condition. By obtaining these observations, in order to explore the potential physiological mechanisms of EE exposure, we next aimed to develop and simulate a computational microcircuit model based on the biologically plausible structure of L2/3 of the visual cortex.

### 3.2 Responses of the cortical microcircuit model to simulations mimicking the SH and EE exposure conditions

To understand the possible mechanism of EE-exposure-induced neuronal modulation in L2/3 of the mouse V1, we performed simulations using a microcircuit model with a biologically plausible L2/3 network of V1 (Figure 2A) (Wagatsuma et al., 2023). The fundamental structure and network of our microcircuit model are described in the Materials and Methods section. This microcircuit model of L2/3 in V1 incorporated excitatory Pyr neurons and three inhibitory interneuron subclasses: PV, SOM, and VIP. In the proposed network, synaptic strengths among Pyr neurons were characterized by a long-tailed, log-normal distribution (Figure 2B) (Song et al., 2005; Lefort et al., 2009; Teramae et al., 2012). Other synaptic connections obeyed a Gaussian distribution (Figure 2C).

In this study, the experimental conditions for SH and EE exposure were distinguished based on the strengths of the feedforward inputs originating from the retina and the feedback signals derived from higher visual areas. Feedforward inputs mediated by AMPA type glutamatergic receptors were applied to all classes and subclasses of the model neuron population, whereas the feedback signals applied by the NMDA receptor-mediating synaptic currents were projected onto the Pyr model neuron and VIP model interneuron populations (Figure 2A). To simulate the SH condition, we applied Poisson neuron populations with frequencies of 10 Hz and 5 Hz as the feedforward inputs and feedback signals, respectively, to our model. To simulate the EE exposure condition, the feedforward and feedback signals were set to 20 Hz and 10 Hz, respectively.

**Figure 4:**
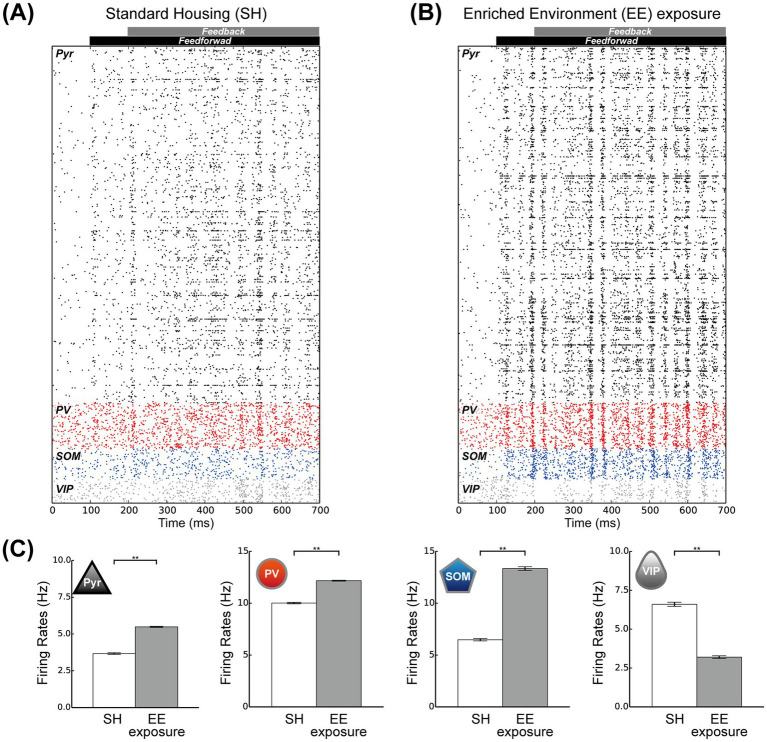
Responses of the proposed microcircuit model. **(A)** Raster plots for the standard housing (SH) condition for all spike trains of the pyramidal (Pyr), parvalbumin (PV), somatostatin (SOM), and vasoactive intestinal polypeptide (VIP) neuron populations for 700 ms. **(B)** Raster plots with respect to the enriched environment (EE) exposure condition for all spike trains of the Pyr, PV, SOM, and VIP neuron populations for 700 ms. **(C)** The mean population firing rates of Pyr, PV, SOM, and VIP neurons for the SH (white) and EE exposure (gray) conditions (10 simulation trials). The error bars indicate the standard error. Asterisks indicate significant differences between the two conditions (*t*-test, ^**^p < 0.01).

Figure 4A shows the spike raster plots of Pyr model neurons and the three subclasses of inhibitory interneurons in the SH condition. In this simulation, we applied feedforward inputs starting at 100 ms and feedback signals starting at 200 ms. These external inputs to the model markedly modulated the activities of all neuron classes and subclasses from the spontaneous condition. The spike raster for the EE exposure condition is presented in Figure 4B. Under this condition, Pyr model neurons and PV and SOM inhibitory interneurons seemed to be more activated than in the SH condition, likely because of the increases in the rates of feedforward inputs and feedback signals. In addition, the responses of the microcircuit model with these feedforward inputs and feedback signals remained stable even during prolonged simulation durations.

To analyze the responses of our microcircuit model, we simulated our network model under these two conditions, performing 10 trials of 60 biological seconds for each condition, and computed the population rates for Pyr model neurons and the three subclasses of inhibitory model interneurons (Figure 4C). The population activities of Pyr model neurons and PV and SOM inhibitory model interneurons were significantly enhanced under the EE exposure condition compared with those under the SH condition (*t*-test, *p* < 0.01). Conversely, the population activities of the VIP model interneuron subclass significantly decreased from the SH to the EE exposure condition (*t*-test, *p* < 0.01). These results implied that the responses of our microcircuit model are dependent on the strengths of the feedforward inputs and feedback signals. Specifically, enhancement of these external inputs significantly activated the Pyr model neuron population, consistent with the effects of the switch from SH to EE observed in our experiments, as shown in Figure 3A.

**Figure 5:**
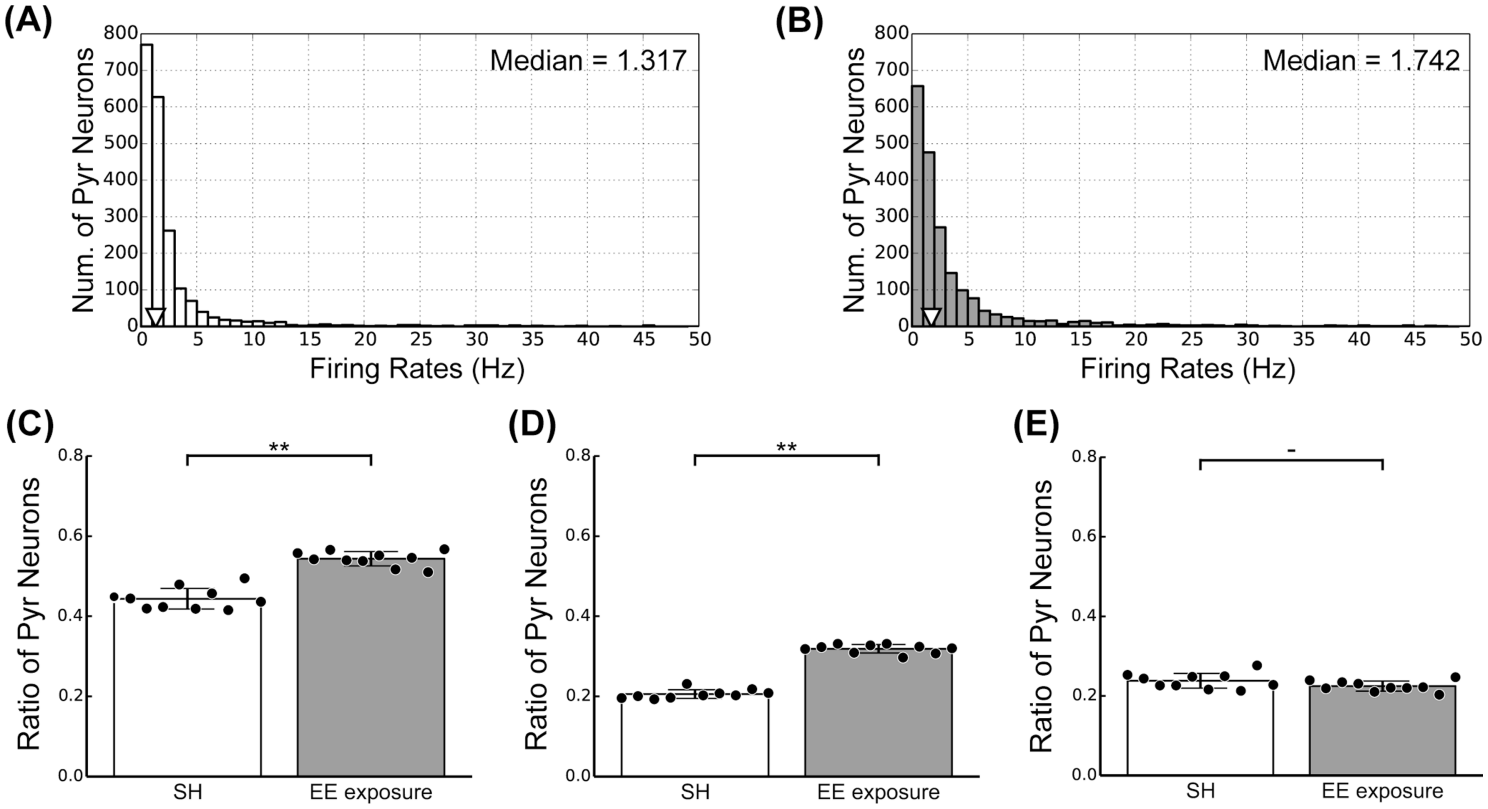
Activity of excitatory pyramidal (Pyr) model neurons in the standard housing (SH) and enriched environment (EE) exposure conditions. **(A)** The distribution of firing rates of Pyr neurons under the SH condition. **(B)** The distribution of firing rates of Pyr neurons under the EE exposure condition. **(C)** Ratio of positive Pyr model neurons with firing rates exceeding 1.5 Hz within the Pyr population. **(D)** Ratio of strong positive Pyr model neurons with firing rates exceeding 3.0 Hz. **(E)** Ratio of weak positive Pyr model neurons with a firing rate ranging from 1.5 to 3.0 Hz. Asterisks indicate significant differences between the two conditions (*t*-test, ^**^p < 0.01, ^-^*p* < 0.1).

Our physiological experiments revealed characteristic modulation patterns in L2/3 of V1 induced by switching from SH to EE (Figure 3). To relate the responses of our microcircuit model to our physiological observations, we computed the firing rates of individual Pyr model neurons under both the SH and EE exposure conditions. The distributions of firing rates of individual Pyr model neurons under the SH and EE exposure conditions are presented in Figures 5A,B, respectively. In the EE exposure condition, enhancements in feedforward inputs and feedback signals resulted in a rightward shift of the median value compared with those in the SH condition, as indicated by triangles in Figures 5A,B. Furthermore, the number of Pyr model neurons with firing rates ranging from 0 to 2 Hz in the EE exposure condition was less than that in the SH condition. These results implied a marked increase in highly activated Pyr model neurons under the EE exposure condition compared with that in the SH condition, and this characteristic is similar to that observed in our physiological observations.

To further analyze the responses of our microcircuit model, we assumed that Pyr model neurons with a firing rate ranging from 1.5 to 3.0 Hz correspond to c-Fos-weak positive cells (weak positive Pyr neurons). The threshold firing rate to distinguish weak positive from inactivated Pyr neurons was based on approximately twice the spontaneous population activity of Pyr neurons (0.6 Hz). In contrast, we classified Pyr model neurons with firing rates exceeding 3.0 Hz as c-Fos-strong positive cells (strong positive Pyr neurons). We computed the ratios of weak and strong positive Pyr neurons within the Pyr model neuron population to compare physiological activities in L2/3 of the mouse V1, as shown in Figure 3, with the responses generated by our model. The ratio of positive Pyr model neurons with firing rates exceeding 1.5 Hz within the Pyr population is presented in Figure 5C. Furthermore, we display the ratios of strong and weak positive Pyr neurons within the Pyr model neuron population in Figures 5D,E, respectively. The EE exposure condition significantly increased the ratio of positive Pyr model neurons, as well as the ratio of strong positive Pyr model neurons, compared with the SH condition (*t*-test, *p* < 0.01; Figures 5C,D). However, there was no significant difference in the ratio of weak positive Pyr neurons between the SH and EE exposure conditions (*t*-test, *p* = 0.09; Figure 5E). These modulation patterns generated by our model were statistically in good agreement with the patterns of neuronal response observed in our physiological experiments depicted in Figure 3.

The population activities of PV and SOM inhibitory model interneurons, as well as Pyr model neurons, significantly increased from the SH to the EE exposure condition (Figure 4C). To gain deeper insights into the model’s responses, we analyzed the firing rates of individual PV and SOM interneurons under both SH and EE exposure conditions (Supplementary Figures S1A–D). Regardless of the simulation conditions, the firing rate distributions of individual PV and SOM model interneurons exhibited characteristics similar to those observed in the Pyr population (Figures 5A,B). Furthermore, we computed the ratios of weak and strong positive model neurons within the Pyr, PV, and SOM model populations with respect to both SH and EE exposure conditions (Supplementary Figures S1E,G). In these analyses, the firing rate thresholds were consistent with those applied in our previous analyses presented in Figures 5C–E. The modulation patterns observed in the ratios of positive, strong-positive, and weak-positive model neurons within these populations were statistically consistent with those observed in our physiological experiments.

### 3.3 Analyses of the threshold for distinguishing between weak and strong positive Pyr model neurons

We analyzed the ratios of weak and strong positive Pyr neurons within the Pyr model neuron population (Figure 5) to relate the neuronal responses physiologically observed in L2/3 of the mouse V1 (Figure 3). In our analyses, Pyr model neurons with firing rates from 1.5 to 3.0 Hz were classified as c-Fos-weak positive, while those with rates exceeding 3.0 Hz were categorized as c-Fos-strong positive. However, the results of these analyses depended on the threshold set for the firing rate used to distinguish between positive and negative (inactive) Pyr model neurons. To further analyze the responses of our model, we reanalyzed the ratios of weak and strong positive Pyr model neurons while varying the thresholds for weak positive Pyr neurons, with firing rates ranging from 1.0 to 2.0 Hz, 1.5 to 3.0 Hz, and 2.0 to 4.0 Hz, as functions of feedforward and feedback frequencies. For this analysis, we performed 50 simulation trials with a length of 6 biological seconds by systematically adjusting the rates of these two inputs. The means of feedforward inputs and feedback signals were systematically varied in the range from 0 to 30 Hz, in steps of 5 Hz.

**Figure 6:**
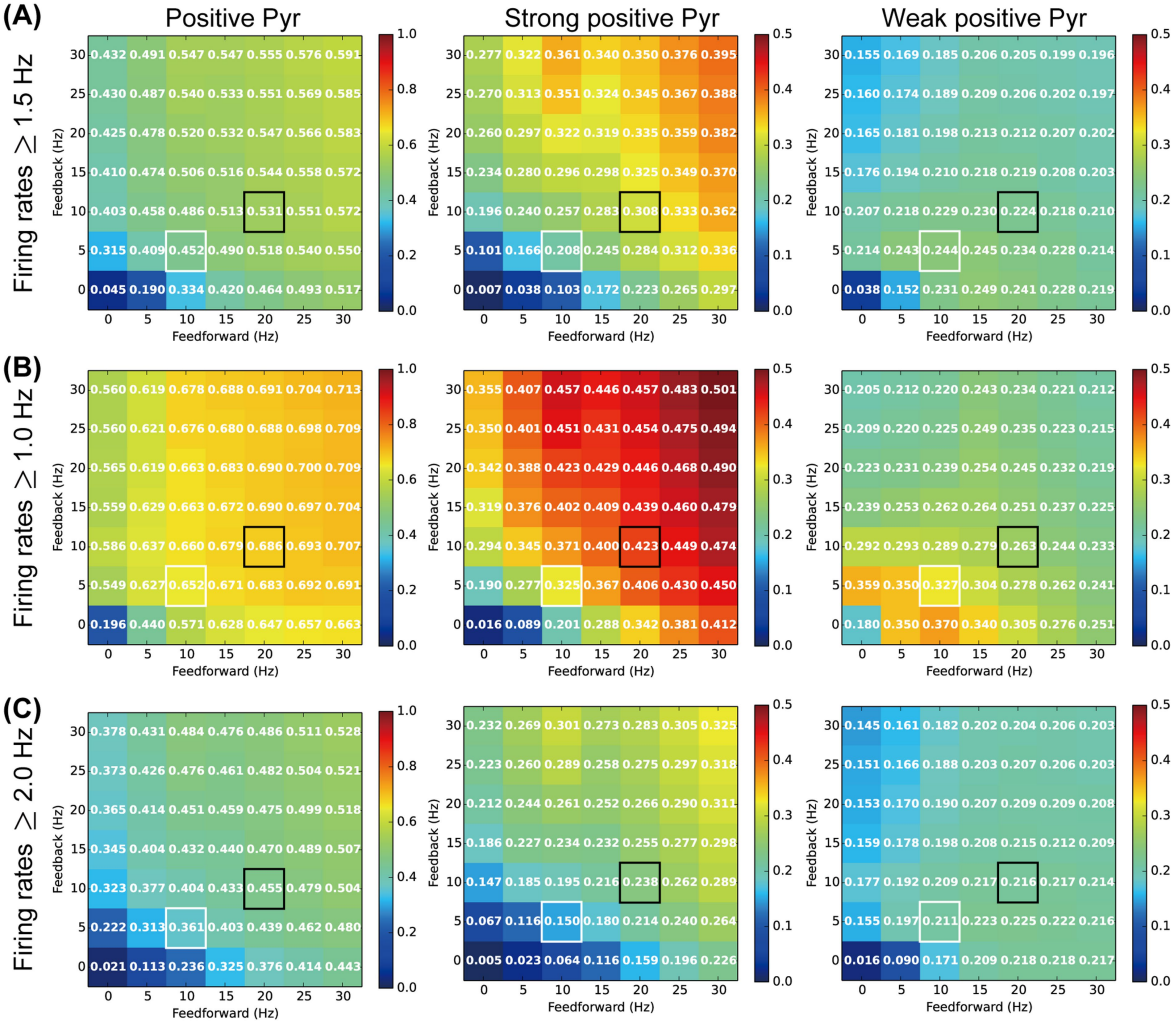
Influences of the inputs and threshold on our analyses. The colors and values of the cells in the matrix represent the ratio of activated pyramidal (Pyr) model neurons. The *x*- and *y*-axes indicate firing rates of feedforward and feedback inputs to our model, respectively. Cells with white and black borders in each panel correspond to the standard housing and enriched environment exposure conditions of our model simulations, respectively. **(A)** Ratio of c-Fos-positive Pyr model neurons based on a firing rate threshold of 1.5 Hz. The left, middle, and right panels indicate the ratios of c-Fos-positive (≥1.5 Hz), strong positive (>3.0 Hz), and weak positive (1.5–3.0 Hz) Pyr model neurons, respectively. **(B)** Ratio of c-Fos positive Pyr model neurons based on a firing rate threshold of 1.0 Hz. The left, middle, and right panels indicate the ratios of c-Fos-positive (≥1.0 Hz), strong positive (>2.0 Hz), and weak positive (1.0–2.0 Hz) Pyr model neurons, respectively. **(C)** Ratio of c-Fos-positive Pyr model neurons based on a firing rate threshold of 2.0 Hz. The left, middle, and right panels indicate ratios of c-Fos-positive (≥2.0 Hz), strong positive (>4.0 Hz), and weak positive (2.0–4.0 Hz) Pyr model neurons, respectively.

Figure 6 summarizes the ratios of positive, strong positive, and weak positive Pyr model neurons within the Pyr neuron population using weak positive thresholds ranging from 1.5 to 3.0 Hz (Figure 6A), 1.0 to 2.0 Hz (Figure 6B), and 2.0 to 4.0 Hz (Figure 6C). These ratios are presented as functions of feedforward and feedback signal frequencies. In each panel of Figure 6, cells outlined with white and black borders represent the ratios from simulations under our SH and EE exposure conditions, respectively. Irrespective of the threshold level, the ratios of positive Pyr model neurons monotonically increased with increases in both feedforward inputs and feedback signals, as shown in the left column of Figure 6. This trend was common for observations of strong positive Pyr model neurons (center column in Figure 6). These results are consistent with the physiologically observed significant increase in the ratios of c-Fos-positive and strong positive neurons in L2/3 of V1 induced by the switch from SH to EE (Figures 3C,F).

The ratios of weak positive Pyr model neurons were also influenced by the feedforward inputs and feedback signals, as well as by the threshold levels (right column in Figure 6). However, modulation of the ratio of weak positive Pyr model neurons by feedforward inputs and feedback signals was more moderate than that observed for the ratio of strong positive Pyr model neurons. In particular, across these three threshold levels, the decrease in the ratio of weak positive Pyr model neurons from the SH (white border) to EE exposure (black border) condition was substantially less than the increase observed in the ratio of strong positive neurons. This trend was consistent with our physiological observation that the switch from SH to EE does not significantly modulate the ratio of c-Fos-weak positive neurons in L2/3 of V1 (Figure 3G).

These results suggest that the ratios of weak and strong positive Pyr model neurons fluctuate depending on the threshold levels. However, the impact of feedforward inputs and feedback signals on the ratio of weak positive Pyr neurons was relatively moderate compared with their effect on strong positive Pyr neurons, particularly when weak positive thresholds were chosen to approximately twice or triple the spontaneous population activity of Pyr model neurons. Additionally, these thresholds appeared to be near the median of the distribution of firing rates in the Pyr model neuron population (Figures 5A,B). The potential mechanisms underlying these modulations, as suggested by our microcircuit model, will be discussed further in the Discussion section.

## 4 Discussion

In this study, we physiologically and computationally investigated the influences of the switch from SH to EE on responses in L2/3 of the mouse V1 (Figure 1). EE modulates plasticity within the visual cortex by inducing the expression of immediate early genes (Montero, 1997; Cancedda et al., 2004; Piché et al., 2004). Our physiological experiments employed immunostaining of the immediate early gene product c-Fos after EE exposure (Sheng and Greenberg, 1990). The switch from SH to EE significantly increased the number of c-Fos-strong positive cells in L2/3 compared with those in the SH condition, whereas there was no significant difference in the number of c-Fos-weak positive cells between these conditions. We should note that the first exposure to EE inevitably involves novelty exposure, and thus the increases in strong c-Fos positive cells could also reflect changes in neuronal activity by novelty exposure. These characteristic EE-exposure-induced modulation patterns observed in L2/3 of the mouse V1 were quantitatively reproduced by the cortical microcircuit model using a biologically plausible network of L2/3 of V1 (Figure 2). These physiological and computational findings not only imply the presence of characteristic modulation of neuronal responses in V1 because of the switch from SH to EE, but also provide insights into the mechanisms underlying neuronal modulation by EE exposure.

### 4.1 The potential mechanism for EE-exposure-induced modulation of the neuronal response of L2/3 of V1

Our physiological experiments indicated an EE-exposure-induced increase in the number of c-Fos-strong positive cells in L2/3 of V1, whereas the ratio of c-Fos-weak positive cells remained constant between the EE exposure and SH conditions (Figure 3). These findings were quantitatively replicated by our model based on recent knowledge of the structure of L2/3 of V1 (Wagatsuma et al., 2023) (Figure 5). In our network model, the synaptic strengths among Pyr model neurons conformed to a log-normal distribution (Figure 2B) (Song et al., 2005; Lefort et al., 2009; Teramae et al., 2012). Additionally, our simulations of the model revealed that the firing rates of individual Pyr model neurons also exhibited a log-normal distribution (Figures 5A,B). In contrast, when the firing rates of Pyr model neurons followed a Gaussian distribution with consistent variance under both the SH and EE exposure conditions, the number of weak positive Pyr model neurons notably decreased from the SH to the EE exposure condition. Our microcircuit model suggested that the log-normally distributed synaptic strengths within the Pyr neuron population underlay the EE-exposure-induced characteristic modulation patterns observed in our physiological experiments.

Our model simulated a single microcircuit responsible for processing visual features within a distinct receptive field. However, many excitatory neurons in the superficial layer of V1 project their signals horizontally and interact with each other over large distances across their receptive fields (Rockland and Pandya, 1979; Gilbert and Wiesel, 1983; Hirsch and Gilbert, 1991), potentially underlying the surrounding modulation observed in the visual cortices (Malach et al., 1993; Veit et al., 2017). The surrounding modulation and interactions through horizontal connections across receptive fields might be notably enhanced under the EE exposure condition compared with those in the SH condition because of the diversity of objects provided to animals exposed to EE (Figure 1). It is possible that these enhanced interactions within V1 across receptive fields play an important role in inducing the characteristic physiological modulations observed with the switch from SH to EE.

### 4.2 Difference between the switch from SH to EE and locomotion effects

Locomotion is an important factor in enhancing the responses of the visual system, including the mouse V1 (Fu et al., 2014; Erisken et al., 2014). It is possible that the observed physiological increase in the number of c-Fos-strong positive cells in L2/3 of the mouse V1 may be attributable to locomotion rather than the switch from SH to EE. However, a prior study showed that EE decreased coupling between visual and motor cortex activity with respect to SH, despite a lack of significant differences in movement distances and mean velocities between the EE and SH conditions (Garbo et al., 2011). This finding suggests that the observed modulation in L2/3 of V1 in our physiological experiments is attributable to the switch from SH to EE rather than locomotion, highlighting the unique mechanisms of EE exposure distinct from those of locomotion. Analyses of the interactions between EE exposure and locomotion might be important to understand the neuronal mechanisms modulating V1 activity. Additionally, behavioral analyses, such as tracking distances and movement velocities, are essential to understand the effects of the switch from SH to EE on neuronal activity.

### 4.3 Difference in the simulation conditions between the switch from SH to EE exposure and selective attention

In our microcircuit model simulations, the EE exposure condition was modeled by simultaneously increasing the firing rates of both feedforward inputs and feedback signals compared to those in the SH condition. These activated external inputs increased the population activities of Pyr model neurons as well as PV and SOM inhibitory interneurons compared to the SH condition. In contrast, the population activity of VIP interneurons was significantly reduced under the EE exposure condition compared to the SH condition (Figure 4C). Previous computational studies investigating the mechanisms underlying attentional modulation in visual cortices have suggested that selective attention, mediated by feedback signals, enhances the activity of Pyr model neurons, PV interneurons, and VIP interneurons, while suppressing the activity of SOM interneurons (Buia and Tiesinga, 2008; Wagatsuma et al., 2022; Wagatsuma et al., 2023). These studies applied markedly enhanced feedback signals to the VIP population to model the selective attention condition. The modulation patterns reported in these previous models differ markedly from those observed in the simulations of our current model. These discrepancies appear to result from differences in the simulation conditions. In these previous models, the strength of the feedforward inputs remained constant during simulations with visual stimuli to analyze how selective attention modulated the model responses to their preferred visual stimuli. In addition, only the rates of feedback signals representing selective attention were increased to simulate the condition where the animal attends to a specific visual stimulus. However, unlike previous computational studies, our current simulations simultaneously increased the firing rates of both feedforward inputs and feedback signals under the EE exposure condition compared to the SH condition. In our simulations, the activation of feedforward inputs from the SH to the EE condition likely enhanced the responses of Pyr neurons as well as PV and SOM interneurons, while simultaneously suppressing VIP interneuron responses through SOM interneuron activation. The activation of the SOM population by increased feedforward inputs from the SH to the EE may precede the activation of VIP and suppress VIP responses, as the rise time of NMDA synapses is slower than that of AMPA synapses. Additionally, feedback signals to the VIP population may fail to function effectively under inhibition from the SOM population, due to the voltage-dependent properties of NMDA synapses. Furthermore, the strength of feedback signals under the EE exposure condition in our current model was markedly weaker than that of the feedback signals representing the selective attention condition reported by Wagatsuma et al. (2023). These variations in external inputs within the cortical microcircuit model may be a critical factor for considering simulation conditions of the model.

### 4.4 Future physiological experiments and further development of the microcircuit model to investigate the effects of the switch from SH to EE

Our physiological experiment indicated that switching from SH to EE significantly increased the number of c-Fos-strong positive cells in L2/3 compared to the SH condition. In contrast, no significant difference was observed in the number of c-Fos-weak positive cells between the two conditions (Figure 3). However, the duration of EE exposure in our experiment may not have been sufficient to comprehensively evaluate the criteria for an enriched environment protocol. The first exposure to EE inherently includes an element of novelty, and therefore, the observed increase in strong c-Fos-positive cells may also reflect changes in neuronal activity induced by novelty exposure. Additionally, previous studies investigating the impacts of EE exposure on the modulation of neuronal responses often exposed animals to enriched environments for longer durations than those used in our experiment (Hüttenrauch et al., 2016; Martínez-Torres et al., 2024; Miranda et al., 2024). Further physiological experiments with extended durations of EE exposure may be necessary to investigate and analyze the impacts of the switch from SH to EE on modulation of neuronal responses in animals.

Prior studies have reported improvements in cognitive and behavioral functions because of EE across both healthy and pathological conditions that involve cognitive decline (Leggio et al., 2005; Nithianantharajah and Hannan, 2006; Nithianantharajah and Hannan, 2009; Petrosini et al., 2009; Hannan, 2014; Mandolesi et al., 2017; Crawford et al., 2020). Particularly, in a rat model of schizophrenia, exposure to EE was shown to enhance performance in tasks involving reversal learning and extradimensional shifting (Saland and Rodefer, 2011). Moreover, EE is often employed in studies exploring dysfunctions across various neural systems, including autism spectrum disorder (ASD) and schizophrenia (Caires and Bossolani-Martins, 2023; Toyoshima et al., 2025). By applying the experimental methods and analyses used in this study to ASD and schizophrenia model mice, it may be possible to provide insights into the effects of EE on these complex and heterogeneous neurodevelopmental/psychiatric disorders and their impact on the perception of affected patients.

In individuals with ASD and schizophrenia, it is postulated that the dysfunction of various neural systems, such as atypical visual perception, originates from an excitatory/inhibitory imbalance in the brain (Driesen et al., 2013; Lisman, 2012; Murray et al., 2014; Uhlhaas and Singer, 2010; Obi-Nagata et al., 2023; Santoro et al., 2024). Additionally, to examine the neural mechanism of the pathological condition, Nobukawa et al. (2022) computationally investigated the influences of excitatory/inhibitory balance disruptions by altering the numbers of excitatory and inhibitory neurons in their model. If incorporating such excitatory/inhibitory balance disruptions into our model enables us to model the network of the L2/3 disorder in V1, it will become possible to simulate the effects of EE on model mouse pathology, as assessed in the experiments in the current study.

## Data Availability

The codes in the study are deposited in the Figshare repository, can be accessed with https://doi.org/10.6084/m9.figshare.20934352.

## References

[ref1] Bibollet-Bahena O. Tissier S. Ho-Tran S. Rojewski A. Casanova C. (2023). Enriched environment exposure during development positively impacts the structure and function of the visual cortex in mice. Sci. Rep. 13:7020. doi: 10.1038/s41598-023-33951-0 37120630 PMC10148800

[ref2] Binzegger T. Douglas R. J. Martin K. A. C. (2004). A quantitative map of the circuit of cat primary visual cortex. J. Neurosci. 24, 8441–8453. doi: 10.1523/JNEUROSCI.1400-04.2004, PMID: 15456817 PMC6729898

[ref3] Buehlmann A. Deco G. (2008). The neural basis of attention: rate versus synchronization modulation. J. Neurosci. 28, 7679–7686. doi: 10.1523/JNEUROSCI.5640-07.2008, PMID: 18650344 PMC6670852

[ref4] Buia C. I. Tiesinga P. H. (2008). Role of interneuron diversity in the cortical microcircuit for attention. J. Neurophysiol. 99, 2158–2182. doi: 10.1152/jn.01004.2007 18287553

[ref5] Caires C. R. S. Bossolani-Martins A. L. (2023). Which form of environmental enrichment is most effective in rodent models of autism? Behav. Process. 211:104915. doi: 10.1016/j.beproc.2023.104915 37451559

[ref6] Cancedda L. Putignano E. Sale A. Viegi A. Berardi N. Maffei L. (2004). Acceleration of visual system development by environmental enrichment. J. Neurosci. 24, 4840–4848. doi: 10.1523/JNEUROSCI.0845-04.2004, PMID: 15152044 PMC6729456

[ref7] Cardin J. A. (2018). Inhibitory interneurons regulate temporal precision and correlations in cortical circuits. Trends Neurosci. 41, 689–700. doi: 10.1016/j.tins.2018.07.015, PMID: 30274604 PMC6173199

[ref8] Crawford L. E. Knouse L. E. Kent M. Vavra D. Harding O. LeServe D.. (2020). Enriched environment exposure accelerates rodent driving skills. Behav. Brain Res. 378:112309. doi: 10.1016/j.bbr.2019.112309 31629004

[ref9] Deco G. Thiele A. (2011). Cholinergic control of cortical network interactions enables feedback-mediated attentional modulation. Eur. J. Neurosci. 34, 146–157. doi: 10.1111/j.1460-9568.2011.07749.x 21692884

[ref10] Driesen N. R. McCarthy G. Bhagwagar Z. Bloch M. Calhoun V. D’Souza D. C.. (2013). Relationship of resting brain hyperconnectivity and schizophrenia-like symptoms produced by the NMDA receptor antagonist ketamine in humans. Mol. Psychiatry 18, 1199–1204. doi: 10.1038/mp.2012.194 23337947 PMC3646075

[ref11] Erisken S. Vaiceliunaite A. Jurjut O. Fiorini M. Katzner S. Busse L. (2014). Effects of locomotion extend throughout the mouse early visual system. Curr. Biol. 24, 2899–2907. doi: 10.1016/j.cub.2014.10.045 25484299

[ref12] Fu Y. Tucciarone J. M. Espinosa J. S. Sheng N. Darcy D. P. Nicoll R. A.. (2014). A cortical circuit for gain control by behavioral state. Cell 156, 1139–1152. doi: 10.1016/j.cell.2014.01.050 24630718 PMC4041382

[ref13] Garbo A. D. Mainardi M. Chillemi S. Maffei L. Caleo M. (2011). Environmental enrichment modulates cortico-cortical interactions in the mouse. PLoS One 6:e25285. doi: 10.1371/journal.pone.0025285 21966482 PMC3178623

[ref14] Gilbert C. D. Wiesel T. N. (1983). Clustered intrinsic connections in cat visual cortex. J. Neurosci. 3, 1116–1133. doi: 10.1523/JNEUROSCI.03-05-01116.1983 6188819 PMC6564507

[ref15] Hannan A. J. (2014). Environmental enrichment and brain repair: harnessing the therapeutic effects of cognitive stimulation and physical activity to enhance experience-dependent plasticity. Neuropathol. Appl. Neurobiol. 40, 13–25. doi: 10.1111/nan.12102 24354721

[ref16] Herrero J. L. Gieselmann M. A. Sanayei M. Thiele A. (2013). Attention-induced variance and noise correlation reduction in macaque V1 is mediated by NMDA receptors. Neuron 78, 729–739. doi: 10.1016/j.neuron.2013.03.029, PMID: 23719166 PMC3748348

[ref17] Hirsch J. A. Gilbert C. D. (1991). Synaptic physiology of horizontal connections in the cat’s visual cortex. J. Neurosci. 11, 1800–1809. doi: 10.1523/JNEUROSCI.11-06-01800.1991, PMID: 1675266 PMC6575415

[ref18] Hubel D. H. Wiesel T. N. (1968). Receptive fields and functional architecture of monkey striate cortex. J. Physiol. 195, 215–243. doi: 10.1113/jphysiol.1968.sp008455, PMID: 4966457 PMC1557912

[ref19] Hüttenrauch M. Salinas G. Wirths O. (2016). Effects of long-term environmental enrichment on anxiety, memory, hippocampal plasticity and overall brain gene expression in C57BL6 mice. Front. Mol. Neurosci. 9:62. doi: 10.3389/fnmol.2016.00062 27536216 PMC4971077

[ref20] Ishikawa A. W. Komatsu Y. Yoshimura Y. (2014). Experience-dependent emergence of fine-scale networks in visual cortex. J. Neurosci. 34, 12576–12586. doi: 10.1523/JNEUROSCI.1346-14.2014, PMID: 25209295 PMC6615498

[ref21] Ito S. Piet A. Bennett C. Durand S. Belski H. Garrett M.. (2024). Coordinated changes in a cortical circuit sculpt effects of novelty on neural dynamics. Cell Rep. 43:114763. doi: 10.1016/j.celrep.2024.114763, PMID: 39288028 PMC11563561

[ref22] Jahr C. E. Stevens C. F. (1990). Voltage dependence of NMDA-activated macroscopic conductances predicted by single-channel kinetics. J. Neurosci. 10, 3178–3182. doi: 10.1523/JNEUROSCI.10-09-03178.1990, PMID: 1697902 PMC6570236

[ref23] Jenks K. R. Shepherd J. D. (2020). Experience-dependent development and maintenance of binocular neurons in the mouse visual cortex. Cell Rep. 30, 1982–1994.e4. doi: 10.1016/j.celrep.2020.01.031 32049025 PMC7041998

[ref24] Jiang H.-J. Qi G. Duarte R. Feldmeyer D. van Albada S. J. (2024). A layered microcircuit model of somatosensory cortex with three interneuron types and cell-type-specific short-term plasticity. Cereb. Cortex 34:bhae378. doi: 10.1093/cercor/bhae378 39344196 PMC11439972

[ref25] Kalogeraki E. Pielecka-Fortuna J. Löwel S. (2017). Environmental enrichment accelerates ocular dominance plasticity in mouse visual cortex whereas transfer to standard cages resulted in a rapid loss of increased plasticity. PLoS One 12:e0186999. doi: 10.1371/journal.pone.0186999 29073219 PMC5658117

[ref26] Kawashima T. Okuno H. Nonaka M. Adachi-Morishima A. Kyo N. Okamura M.. (2009). Synaptic activity-responsive element in the Arc/Arg3.1 promoter essential for synapse-to-nucleus signaling in activated neurons. Proc. Natl. Acad. Sci. U.S.A. 106, 316–321. doi: 10.1073/pnas.0806518106, PMID: 19116276 PMC2629236

[ref27] Kempermann G. (2019). Environmental enrichment, new neurons and the neurobiology of individuality. Nat. Rev. Neurosci. 20, 235–245. doi: 10.1038/s41583-019-0120-x 30723309

[ref28] Lee J. H. Koch C. Mihalas S. (2017). A computational analysis of the function of three inhibitory cell types in contextual visual processing. Front. Comput. Neurosci. 11:28. doi: 10.3389/fncom.2017.00028 28487644 PMC5403882

[ref29] Lee B. Shin D. Gross S. P. Cho K. H. (2018). Combined positive and negative feedback allows modulation of neuronal oscillation frequency during sensory processing. Cell Rep. 25, 1548–1560.e3. doi: 10.1016/j.celrep.2018.10.029, PMID: 30404009

[ref30] Lefort A. Tomm C. Sarria J.-C. F. Petersen C. C. H. (2009). The excitatory neuronal network of the C2 barrel column in mouse primary somatosensory cortex. Neuron 61, 301–316. doi: 10.1016/j.neuron.2008.12.020, PMID: 19186171

[ref31] Leggio M. G. Mandolesi L. Federico F. Spirito F. Ricci B. Gelfo F.. (2005). Environmental enrichment promotes improved spatial abilities and enhanced dendritic growth in the rat. Behav. Brain Res. 163, 78–90. doi: 10.1016/j.bbr.2005.04.009 15913801

[ref32] Lisman J. (2012). Excitation, inhibition, local oscillations, or large-scale loops: what causes the symptoms of schizophrenia? Curr. Opin. Neurobiol. 22, 537–544. doi: 10.1016/j.conb.2011.10.018, PMID: 22079494 PMC3302967

[ref33] Mainardi M. Landi S. Gianfranceschi L. Baldini S. De Pasquale R. Berardi N.. (2010). Environmental enrichment potentiates thalamocortical transmission and plasticity in the adult rat visual cortex. J. Neurosci. Res. 88, 3048–3059. doi: 10.1002/jnr.22461 20722076

[ref34] Malach R. Amir Y. Harel M. Grinvald A. (1993). Relationship between intrinsic connections and functional architecture revealed by optical imaging and in vivo targeted biocytin injections in primate striate cortex. Proc. Natl. Acad. Sci. U.S.A. 90, 10469–10473. doi: 10.1073/pnas.90.22.10469, PMID: 8248133 PMC47798

[ref35] Mandolesi L. Gelfo F. Serra L. Montuori S. Polverino A. Curcio G.. (2017). Environmental factors promoting neural plasticity: insights from animal and human studies. Neural Plast. 2017:19461. doi: 10.1155/2017/7219461 PMC5504954 28740740

[ref36] Mardinly A. R. Spiegel I. Patrizi A. Centofante E. Bazinet J. E. Tzeng C. P.. (2016). Sensory experience regulates cortical inhibitory by inducing IGF1 in VIP neurons. Nature 531, 371–375. doi: 10.1038/nature17187, PMID: 26958833 PMC4823817

[ref37] Martínez-Torres N. I. Cárdenas-Bedoya J. Vázquez-Torres B. M. Torres-Mendoza B. M. (2024). Environmental enrichment and cerebrolysin improve motor and cognitive performance in a rat model of stroke, in conjunction with an increase in hippocampal AMPA but not NMDA receptor subunits. Brain Res. 1825:148694. doi: 10.1016/j.brainres.2023.148694 38048977

[ref38] Miranda M. Navas M. C. Saad M. B. Z. Girado D. P. Weisstaub N. Bekinschtein P. (2024). Environmental enrichment in middle age rats improves spatial and object memory discrimination deficits. Front. Behav. Neurosci. 18:1478656. doi: 10.3389/fnbeh.2024.1478656 39494036 PMC11528545

[ref39] Montero V. M. (1997). c-Fos induction in sensory pathways of rats exploring a novel complex environment: shifts of active thalamic reticular sectors by predominant sensory cues. Neuroscience 76, 1069–1081. doi: 10.1016/s0306-4522(96)00417-4, PMID: 9027867

[ref40] Morgan J. I. Curran T. (1991). Stimulus-transcription coupling in the nervous system: involvement of the inducible proto-oncogenes fos and jun. Annu. Rev. Neurosci. 14, 421–451. doi: 10.1146/annurev.ne.14.030191.002225, PMID: 1903243

[ref41] Murray J. D. Anticevic A. Gancsos M. Ichinose M. Corlett P. R. Krystal J. H.. (2014). Linking microcircuit dysfunction to cognitive impairment: effects of disinhibition associated with schizophrenia in a cortical working memory model. Cereb. Cortex 24, 859–872. doi: 10.1093/cercor/bhs370 23203979 PMC3948492

[ref42] Neske G. T. Patrick S. L. Connor B. W. (2015). Contributions of diverse excitatory and inhibitory neurons to recurrent network activity in cerebral cortex. J. Neurosci. 35, 1089–1105. doi: 10.1523/JNEUROSCI.2279-14.2015 25609625 PMC4300319

[ref43] Nguyen H. N. Huppé-Gourgues H. Vaucher E. (2015). Activation of the mouse primary visual cortex by medial prefrontal subregion stimulation is not mediated by cholinergic basalo-cortical projections. Front. Syst. Neurosci. 9:1. doi: 10.3389/fnsys.2015.00001 25709570 PMC4321436

[ref44] Nithianantharajah J. Hannan A. J. (2006). Enriched environments, experience-dependent plasticity and disorders of the nervous system. Nat. Rev. Neurosci. 7, 697–709. doi: 10.1038/nrn1970 16924259

[ref45] Nithianantharajah J. Hannan A. J. (2009). The neurobiology of brain and cognitive reserve: mental and physical activity as modulators of brain disorders. Prog. Neurobiol. 89, 369–382. doi: 10.1016/j.pneurobio.2009.10.001, PMID: 19819293

[ref46] Nobukawa S. Wagatsuma N. Ikeda T. Hasegawa C. Kikuchi M. Takahashi T. (2022). Effect of steady-state response versus excitatory/inhibitory balance on spiking synchronization in neural networks with log-normal synaptic weight distribution. Cogn. Neurodyn. 16, 871–885. doi: 10.1007/s11571-021-09757-z 35847535 PMC9279535

[ref47] Obi-Nagata K. Suzuki N. Miyake R. MacDonald M. L. Fish K. N. Ozawa K.. (2023). Distorted neurocomputation by a small number of extra-large spines in psychiatric disorders. Sci. Adv. 9:eade5973. doi: 10.1126/sciadv.ade5973 37294752 PMC10256173

[ref48] Pasupathy A. Connor C. E. (2002). Population coding of shape in area V4. Nat. Neurosci. 5, 1332–1338. doi: 10.1038/nn972 12426571

[ref49] Petrosini L. De Bartolo P. Foti F. Gelfo F. Cutuli D. Leggio M. G.. (2009). On whether the environmental enrichment may provide cognitive and brain reserves. Brain Res. Rev. 61, 221–239. doi: 10.1016/j.brainresrev.2009.07.002 19631687

[ref50] Pfeffer C. K. Xue M. He M. Huang Z. J. Scanziani M. (2013). Inhibition of inhibition in visual cortex: the logic of connections between molecularly distinct interneurons. Nat. Neurosci. 16, 1068–1076. doi: 10.1038/nn.3446, PMID: 23817549 PMC3729586

[ref51] Piché M. Robert S. Miceli D. Bronchti G. (2004). Environmental enrichment enhances auditory takeover of the occipital cortex in anophthalmic mice. Eur. J. Neurosci. 20, 3463–3472. doi: 10.1111/j.1460-9568.2004.03823.x, PMID: 15610179

[ref52] Pizzo R. Gurgone A. Castroflorio E. Amendola E. Gross C. Sassoè-Pognetto M.. (2016). Lack of Cdkl5 disrupts the organization of excitatory and inhibitory synapses and parvalbumin interneurons in the primary visual cortex. Front. Cell. Neurosci. 10:261. doi: 10.3389/fncel.2016.00261, PMID: 27965538 PMC5124713

[ref53] Pompeiano M. Colonnese M. T. (2023). cFOS as a biomarker of activity maturation in the hippocampal formation. Front. Neurosci. 17:929461. doi: 10.3389/fnins.2023.929461 37521697 PMC10374841

[ref54] Potjans T. C. Diesmann M. (2014). The cell-type specific cortical microcircuit: relating structure and activity in a full-scale spiking network model. Cereb. Cortex 24, 785–806. doi: 10.1093/cercor/bhs358 23203991 PMC3920768

[ref55] Rockland K. S. Pandya D. N. (1979). Laminar origins and terminations of cortical connections of the occipital lobe in the rhesus monkey. Brain Res. 179, 3–20. doi: 10.1016/0006-8993(79)90485-2 116716

[ref56] Saland S. K. Rodefer J. S. (2011). Environmental enrichment ameliorates phencyclidine-induced cognitive deficits. Pharmacol. Biochem. Behav. 98, 455–461. doi: 10.1016/j.pbb.2011.02.014 21334364

[ref57] Sale A. Berardi N. Maffei L. (2009). Enrich the environment to empower the brain. Trends Neurosci. 32, 233–239. doi: 10.1016/j.tins.2008.12.004 19268375

[ref58] Sampedro-Piquero P. Zancada-Menendez C. Begega A. (2015). Housing condition-related changes involved in reversal learning and its c-Fos associated activity in the prefrontal cortex. Neuroscience 307, 14–25. doi: 10.1016/j.neuroscience.2015.08.038, PMID: 26314630

[ref59] Santoro V. Hou M. D. Premoli I. Belardinelli P. Biondi A. Carobin A.. (2024). Investigating cortical excitability and inhibition in patients with schizophrenia: a TMS-EEG study. Brain Res. Bull. 212:110972. doi: 10.1016/j.brainresbull.2024.110972 38710310

[ref60] Schmehl M. N. Caruso V. C. Chen Y. Jun N. Y. Willett S. M. Mohl J. T.. (2024). Multiple objects evoke fluctuating responses in several regions of the visual pathway. eLife 13:e91129. doi: 10.7554/eLife.91129 38489224 PMC10942787

[ref61] Self M. W. Kooijmans R. N. Super H. Lamme V. A. Roelfsema P. R. (2012). Different glutamate receptors convey feedforward and recurrent processing in macaque V1. Proc. Natl. Acad. Sci. U.S.A. 109, 11031–11036. doi: 10.1073/pnas.1119527109, PMID: 22615394 PMC3390882

[ref62] Self M. W. van Kerkoerle T. Super H. Roelfsema P. R. (2013). Distinct roles of the cortical layers of area V1 in figure-ground segregation. Curr. Biol. 23, 2121–2129. doi: 10.1016/j.cub.2013.09.013 24139742

[ref63] Sheng M. Greenberg M. E. (1990). The regulation and function of c-Fos and other immediate early genes in the nervous system. Neuron 4, 477–485. doi: 10.1016/0896-6273(90)90106-p, PMID: 1969743

[ref64] Song S. Sjöström P. J. Reigl M. Nelson S. Chklovskii D. B. (2005). Highly nonrandom features of synaptic connectivity in local cortical circuits. PLoS Biol. 3:e68. doi: 10.1371/journal.pbio.0030068 15737062 PMC1054880

[ref65] Teramae J. Tsubo Y. Fukai T. (2012). Optimal spike-based communication in excitable networks with strong-sparse and weak-dense links. Sci. Rep. 2:485. doi: 10.1038/srep00485 22761993 PMC3387577

[ref66] Thomson A. M. Morris O. T. (2002). Selectivity in the inter-laminar connections made by neocortical neurons. J. Neurocytol. 31, 239–246. doi: 10.1023/A:1024117908539 12815243

[ref67] Thomson A. M. West D. C. Wang Y. Bannister A. P. (2002). Synaptic connections and small circuits involving excitatory and inhibitory neurons in layers 2–5 of adult rat and cat neocortex: triple intracellular recordings and biocytin labelling in vitro. Cereb. Cortex 12, 936–953. doi: 10.1093/cercor/12.9.936 12183393

[ref68] Toyoshima M. Takahashi K. Sato E. Shimoda S. Yamada K. (2025). Two distinct enriched housings differentially ameliorate object and place recognition deficits in a rat model of schizophrenia. Behav. Brain Res. 476:115276. doi: 10.1016/j.bbr.2024.115276, PMID: 39366555

[ref69] Uhlhaas P. J. Singer W. (2010). Abnormal neural oscillations and synchrony in schizophrenia. Nat. Rev. Neurosci. 11, 100–113. doi: 10.1038/nrn2774 20087360

[ref70] van Kerkoerle T. Self M. W. Dagnino B. Gariel-Mathis M.-A. Poort J. van der Togt C.. (2014). Alpha and gamma oscillations characterize feedback and feedforward processing in monkey visual cortex. Proc. Natl. Acad. Sci. U.S.A. 111, 14332–14341. doi: 10.1073/pnas.1402773111, PMID: 25205811 PMC4210002

[ref71] van Praag H. Kempermann G. Gage F. H. (2020). Neural consequences of environmental enrichment. Nat. Rev. Neurosci. 1, 191–198. doi: 10.1038/35044558, PMID: 11257907

[ref72] Veit J. Hakim R. Jadi M. P. Sejnowski T. J. Adesnik H. (2017). Cortical gamma band synchronization through somatostatin interneurons. Nat. Neurosci. 20, 951–959. doi: 10.1038/nn.4562 28481348 PMC5511041

[ref73] Wagatsuma N. Hu B. von der Heydt R. Niebur E. (2021). Analysis of spiking synchrony in visual cortex reveals distinct types of top-down modulation signals for spatial and object-based attention. PLoS Comput. Biol. 17:e1008829. doi: 10.1371/journal.pcbi.1008829, PMID: 33765007 PMC8023487

[ref74] Wagatsuma N. Nobukawa S. Fukai T. (2023). A microcircuit model involving parvalbumin, somatostatin, and vasoactive intestinal polypeptide inhibitory interneurons for the modulation of neuronal oscillation during visual processing. Cereb. Cortex 33, 4459–4477. doi: 10.1093/cercor/bhac355, PMID: 36130096 PMC10110453

[ref75] Wagatsuma N. Shimomura S. Nobukawa S. (2022). Disinhibitory circuit mediated by connections from vasoactive intestinal polypeptide to somatostatin interneurons underlies the paradoxical decrease in spike synchrony with increased border ownership selective neuron firing rate. Front. Comput. Neurosci. 16:988715. doi: 10.3389/fncom.2022.988715, PMID: 36405781 PMC9672816

[ref76] Wagatsuma N. von der Heydt R. Niebur E. (2016). Spike synchrony generated by modulatory common input through NMDA-type synapses. J. Neurophysiol. 116, 1418–1433. doi: 10.1152/jn.01142.2015 27486111 PMC5040377

[ref77] Wang X. J. (1999). Synaptic basis of cortical persistent activity: the importance of NMDA receptors to working memory. J. Neurosci. 19, 9587–9603. doi: 10.1523/JNEUROSCI.19-21-09587.1999 10531461 PMC6782911

[ref78] Zangenehpour S. Chaudhuri A. (2002). Differential induction and decay curves of c-Fos and zif268 revealed through dual activity maps. Brain Res. Mol. Brain Res. 109, 221–225. doi: 10.1016/s0169-328x(02)00556-9 12531532

[ref79] Zhang S. Xu M. Kamigaki T. Hoang Do J. P. Chang W.-C. Jenvay S.. (2014). Long-range and local circuits for top-down modulation of visual cortex processing. Science 345, 660–665. doi: 10.1126/science.1254126, PMID: 25104383 PMC5776147

[ref80] Zhou H. Friedman H. S. von der Heydt R. (2000). Coding of border ownership in monkey visual cortex. J. Neurosci. 20, 6594–6611. doi: 10.1523/JNEUROSCI.20-17-06594.2000, PMID: 10964965 PMC4784717

