# Supplementary material for: Local connections among excitatory neurons underlie characteristics of enriched environment exposure-induced neuronal response modulation in layers 2/3 of the mouse V1
Source: Front Syst Neurosci. 2025 Feb 19;19:1525717. doi: 10.3389/fnsys.2025.1525717 (PMC11880011; doi:10.3389/fnsys.2025.1525717)
Supplement: Supplementary file 1 [file Data_Sheet_1.pdf]

Supplementary Material

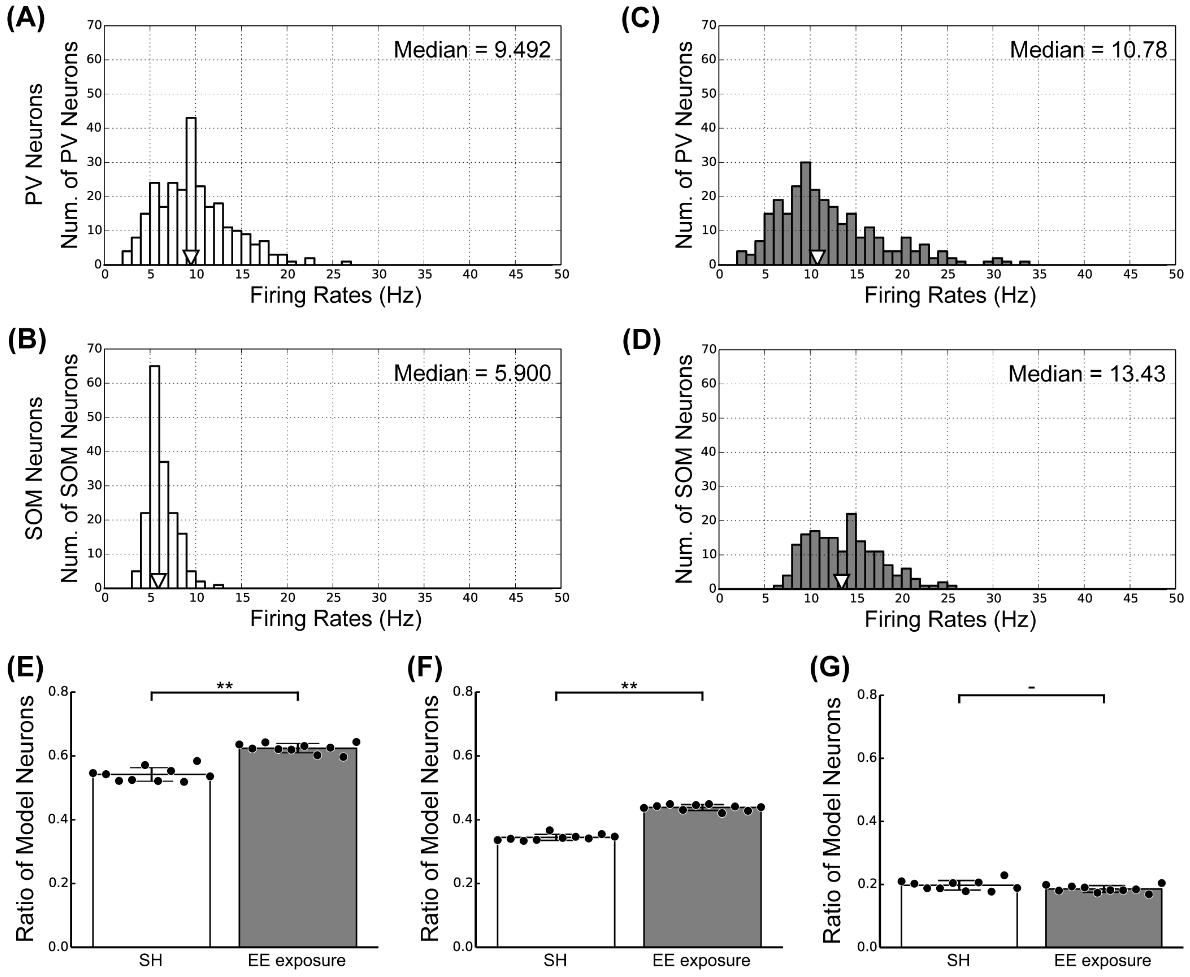

Supplementary Figure 1. Activity of parvalbumin (PV) and somatostatin (SOM) model interneurons in the standard housing (SH) and enriched environment (EE) exposure conditions. ***(A)*** The distribution of firing rates of PV interneurons under the SH condition. ***(B)*** The distribution of firing rates of SOM interneurons under the SH exposure condition. ***(C)*** The distribution of firing rates of PV interneurons under the EE condition. ***(D)*** The distribution of firing rates of SOM interneurons under the EE exposure condition. ***(E)*** Ratio of positive Pyr model neurons, PV and SOM model interneurons with firing rates exceeding 1.5 Hz within these three populations. ***(F)*** Ratio of strong positive Pyr model neurons, PV and SOM model interneurons with firing rates exceeding 3.0 Hz. ***(G)*** Ratio of weak positive Pyr model neurons, PV and SOM model interneurons with a firing rate ranging from 1.5 to 3.0 Hz. Asterisks indicate significant differences between the two conditions (t-test, ** $p < 0.01$, - $p < 0.1$).
